# EEG sensorimotor correlates of translating sounds into actions

**DOI:** 10.3389/fnins.2013.00203

**Published:** 2013-12-11

**Authors:** Jaime A. Pineda, Mark Grichanik, Vanessa Williams, Michelle Trieu, Hailey Chang, Christian Keysers

**Affiliations:** ^1^Department of Cognitive Science, University of CaliforniaSan Diego, La Jolla, CA, USA; ^2^Neurosciences Group, University of CaliforniaSan Diego, La Jolla, CA, USA; ^3^Netherlands Institute for Neuroscience, KNAWAmsterdam, Netherlands; ^4^Department of Neuroscience, University Medical Center Groningen, University of GroningenNetherlands

**Keywords:** auditory mirror neuron system, action comprehension, mu rhythm, sensorimotor cortex, mu suppression

## Abstract

Understanding the actions of others is a necessary foundational cornerstone for effective and affective social interactions. Such understanding may result from a mapping of observed actions as well as heard sounds onto one's own motor representations of those events. To examine the electrophysiological basis of action-related sounds, EEG data were collected in two studies from adults who were exposed to auditory events in one of three categories: action (either hand- or mouth-based sounds), non-action (environmental sounds), and control sounds (scrambled versions of action sounds). In both studies, triplets of sounds of the same category were typically presented, although occasionally, to ensure an attentive state, trials containing a sound from a different category were presented within the triplet and participants were asked to respond to this oddball event either covertly in one study or overtly in another. Additionally, participants in both studies were asked to mimic hand- and mouth-based motor actions associated with the sounds (motor task). Action sounds elicited larger EEG mu rhythm (8–13 Hz) suppression, relative to control sounds, primarily over left hemisphere, while non-action sounds showed larger mu suppression primarily over right hemisphere. Furthermore, hand-based sounds elicited greater mu suppression over the hand area in sensorimotor cortex compared to mouth-based sounds. These patterns of mu suppression across cortical regions to different categories of sounds and to effector-specific sounds suggest differential engagement of a mirroring system in the human brain when processing sounds.

## Introduction

The discovery of motor neurons in the primate premotor cortex that also exhibit visual “mirroring” properties has spurred a significant amount of research into how we understand the actions of others both within and across species having similar biological effectors (di Pellegrino et al., 1992; Rizzolatti and Craighero, 2004; Iacoboni and Dapretto, 2006). Such visuomotor neurons fire both when a monkey performs a motor action and when it observes another conspecific or human agent perform a similar goal-directed action (Rizzolatti and Craighero, 2004; Iacoboni and Dapretto, 2006; Rizzolatti and Sinigaglia, 2010). Evidence for the existence of a human mirror neuron system (MNS) has been obtained through a variety of indirect population-level measures (Iacoboni et al., 1999; Fadiga et al., 2005) including transcranial magnetic stimulation (TMS) (Fadiga et al., 1999; Maeda et al., 2002), positron emission tomography (PET) (Parsons et al., 1995), functional magnetic resonance imaging (fMRI) (Grezes et al., 2003; Buccino et al., 2004; Iacoboni and Dapretto, 2006), and electroencephalography (EEG) (Cochin et al., 1998, 1999; Pineda et al., 2000; Muthukumaraswamy and Johnson, 2004; Muthukumaraswamy and Singh, 2008; Oberman et al., 2008), and is thought to include premotor cortices (dorsal and ventral) and the inferior parietal cortex in which mirror neurons have also been measured in monkeys (Keysers and Gazzola, 2009; Caspers et al., 2010; Rizzolatti and Sinigaglia, 2010), with more recent evidence also pointing to an important role for the somatosensory cortex in the MNS (Keysers et al., 2010; Caspers et al., 2011). While no overall consensus exists as to the role of mirror neurons in social cognition (Hickok, 2009), one prominent hypothesis suggests that the observer's ability to embody the observed action as his or her own provides a neural scaffolding that facilitates behaviors and cognitive outcomes involved in social cognition, such as understanding actions, imitation, speech and language, theory of mind, social communication, and empathy (Rizzolatti and Craighero, 2004; Ferrari et al., 2009).

In the monkey premotor cortex, mirror neurons were also found to be sensitive to the acoustic correlates of actions, and the corresponding action sound by itself is sufficient to activate these premotor cells (Kohler et al., 2002; Keysers et al., 2003). Similarly, the premotor, posterior parietal and somatosensory cortices of humans show voxels that are active both while performing an action and listening to a similar action (Gazzola et al., 2006), and this activity is somatotopically organized, with more dorsal aspects of the premotor and parietal cortex more active during the execution and sound of hand actions, and more ventral aspects more active during the execution and sound of mouth actions. This somatotopical pattern allows classification of what action someone has performed by using the activity pattern while listening to actions (Etzel et al., 2008). Thus, by extension, the human MNS would appear to be multimodal, i.e., activated by motor, visual, auditory, as well as perhaps other sensory inputs associated with the action (Aglioti and Pazzaglia, 2010). Relatively few studies, however, have investigated the auditory properties of the human MNS, although it has been argued that non-action (environmental) and action-related sounds (those that are reproducible by the body) are likely processed by separate neural systems (Pizzamiglio et al., 2005). Non-action related sounds appear to involve the temporal poles, while action-related sounds appear to involve the same neural machinery as the visual MNS. Because increasing the exposure and proficiency with a given physical action provides for greater activation of auditory mirroring circuits (Ricciardi et al., 2009) and a perceptual-motor link that is rapidly established (Lahav et al., 2007), it has been proposed that these associations result from the Hebbian association between motor programs and what they sound like while we perform an action. Re-afference, input that results from the agent's movement, ensures that premotor neurons that cause the action will have firing that is temporally correlated with that of the auditory neurons that represent the re-afferent sound of the action (Keysers and Perrett, 2004). Perhaps one main difference between auditory and visual mirroring is that while visual aspects of mirroring appears to involve bilateral activity in left and right hemispheres, auditory aspects of mirroring has been reported to be primarily left-lateralized, particularly in the parietal cortex, including the posterior parietal and somatosensory cortex (Gazzola et al., 2006; Lahav et al., 2007). This is not true for environmental sounds, that do trigger robust right hemispheric activations as well (Gazzola et al., 2006). The left lateralization may reflect semantic associations of the sounds that have been previously established.

Mirroring activity cannot be directly recorded in humans except under special circumstances, (see Mukamel et al., 2010), but a number of recent studies have suggested that mirroring may be indirectly measured in the mu frequency band of the EEG (alpha: 8–13 Hz and beta: 15–25 Hz recorded over sensorimotor cortex) (Hari et al., 2000; Muthukumaraswamy et al., 2004; Oberman et al., 2005; Pineda, 2005). Sensorimotor neurons fire synchronously at rest, leading to high-amplitude mu oscillations, and asynchronously during self-movement and the observation of movement, leading to reduced amplitude of the mu band (called mu suppression or event related desynchronization-ERD) (Pineda, 2005). Mu suppression or ERD during action observation, in the absence of self-performed action, has been hypothesized to reflect the downstream modulation of sensorimotor neurons by premotor mirror neurons (Muthukumaraswamy et al., 2004; Oberman et al., 2005; Pineda, 2005). A recent combined EEG-fMRI study has explored the relationship between mu suppression (as measured through EEG) and blood oxygen level dependent (BOLD) activity in regions normally associated with the MNS (as measured using fMRI) during both action observation and action execution (Arnstein et al., 2011). The study found that mu suppression in the alpha band during both action observation and action execution went hand-in-hand with increases in BOLD activity in the dorsal premotor cortex, the inferior parietal lobe and the posterior aspects of the somatosensory cortex (BA2). A weaker association was also found between activity in the ventral premotor cortex and mu suppression. All of these regions are associated with the MNS and have strong cortico-cortico connections in the human and non-human brain with the primary sensorimotor region lining the central sulcus (BA4 and BA3) where the mu rhythm is thought to be generated (Shimazu et al., 2004). Therefore, it is plausible that activity in these MNS regions, during action observation and execution, could desynchronize activity around the central sulcus, and thereby cause the mu suppression in the EEG signal. Furthermore, results of several human mu suppression studies parallel primate single-cell recordings in terms of the object-directedness and sensitivity of the electrophysiology to action observation (Muthukumaraswamy et al., 2004). Consistent with this hypothesis, Keuken et al (Keuken et al., 2011) recently showed that using TMS to disrupt activity in the inferior frontal gyrus directly impacts the modulation of mu rhythms over sensorimotor cortex.

To date no investigations have examined the relationship between auditory aspects of mirroring and EEG mu rhythm suppression in humans in order to inform models of connectivity between these domains. One goal of this study was to test the hypothesis that action-related sounds are processed differently compared to non-action related sounds, as reflected in mu rhythm oscillations. We will specifically assess whether mu rhythm suppression reflects action or non-action related activity. In Studies 1 and 2 participants listened to sounds as well as performed actions while blindfolded that corresponded to those sounds. During the active listening portion of Study 1, participants made overt physical responses to oddball sounds. To assess responses to sounds alone, participants made covert responses in Study 2. In both studies, we predicted that representation of action-based sounds would elicit greater mu-suppression reflecting greater engagement of mirroring processes compared to environmental sounds. That is, we expected that action-related sounds (those interpreted vis-a-vis the observer's own bodily representation) would cause greater mu suppression compared to non-action related sounds.

## Materials and methods

### Participants

Twenty-eight healthy undergraduate students, including one older student (12 males and 16 females of varied ethnicities; mean age = 20.3 ± 6.2 years; range = 17–47 years) attending the University of California, San Diego (UCSD) participated in Study 1. In Study 2, a different group of twenty eight undergraduate students (13 males and 15 females; mean age = 20.4 ± 1.1 years; range = 18–23 years) participated. All participants were assumed to have normal hearing if they were able to identify the stimuli during an initial auditory identification task. Those who described themselves as left-handed on a self-report questionnaire were excluded from the study. All gave written informed consent prior to taking part and were compensated with course credit for their voluntary participation. The experiment was reviewed and approved by the UCSD Internal Review Board.

### Auditory stimuli

The auditory stimulus set was obtained from one of the co-authors (C.K.) who previously used it in an fMRI study (Gazzola et al., 2006) that investigated the human auditory MNS. The stimulus set consisted of three categories of sounds (see Table 1): Action (mouth- and hand-based sounds), Non-Action (environmental sounds), and Control or “Fuzzy” (phase-scrambled versions of both mouth and hand control sounds). There were five unique sounds per each category (25 total sounds with each sound presented for 4 s). The Action sounds (e.g., mouth: crunching candy and hand: ripping paper) were tangible sounds that could be easily reproduced by the listener. The Non-Action sounds were comprised of environmental sounds (e.g., howling wind or water dripping). Each control sound was based on one of the action sounds, and resulted from a reverse Fourier transform in which frequencies up to 125 Hz preserved their original phase and all frequencies above 125 Hz had their phase exchanged with that of another frequency. Accordingly, the control sounds were equivalent with respect to the bottom-up global frequency composition of the Action sounds, but were perceived as “fuzzy” because they were phase-scrambled and unrecognizable. For more information on how the sounds were developed see Gazzolla et al. (Gazzola et al., 2006).

**Table 1:** **Auditory stimuli used for Studies 1 and 2**.

**SOUND CATEGORY**	**DESCRIPTION**
Mouth	Crunching a hard candy
	Kissing
	Gargling
	Crunching chips
	Finishing a beverage with a straw
Hand	Ripping a sheet of paper
	Unrolling scotch tape
	Zipping a zipper
	Opening a soda can
	Crushing a soda can
Environmental	Train passing by
	Howling wind
	Waves breaking on the shore
	Electric discharge
	Water dripping
Hand control (Fuzzy)	Crumple can
	Open can
	Zip
	Scotch
	Paper
Mouth control (Fuzzy)	Kiss
	Candy
	Chips
	Gargle
	Straw

### General experimental procedure

All participants took part in an auditory task first and a motor task second. The auditory task was always run before the motor task in order to avoid the possibility that the memory of executing the actions would bias perceptual brain activity. Participants initially completed a general screening questionnaire that enabled the experimenter to exclude individuals due to claustrophobia, prior experience, etc. Prior to EEG recording, participants took part in an Auditory Identification Task in which they were asked to listen attentively and identify the various auditory stimuli. Prior to the presentation of a sound, the experimenter announced the category to which the sound belonged (e.g., Hand Sound). If after three attempts, the participant incorrectly identified the sound, the experimenter would correctly identify the sound for the participant. The sound was played once more before moving on to the next sound to ensure participants now could identify the sound. Participants were not asked to identify nor discriminate control sounds. The experimenter played one of these control sounds and explained that they belonged in their own category of “fuzzy sounds” and did not need to be identified. Prior to the motor task, participants “practiced” performing the motor actions at least once following an experimenter's cue to make sure they followed the protocol.

### EEG recording

Participants were instructed not to consume caffeine nor use any hair products the day of the experiment. During EEG capping, they were seated in a comfortable recliner chair inside an acoustically- and electromagnetically-shielded chamber. After light abrasion of EEG electrode sites using NuPrep Gel, disk electrodes were applied using 10–20 conductive paste on the orbital bone below the left eye and the mastoid bone behind both ears. The eye electrode was used to monitor eye blinks and horizontal eye movements. The mastoid electrodes were computationally linked and used as reference electrodes. Seventeen electrodes embedded in a cap were positioned using the International 10–20 system at the following sites: F7, F8, F3, F4, FZ, C3, CZ, C4, P3, PZ, P4, T3, T4, T5, T6, O1, O2. Electrolytic gel was injected at each electrode site and the scalp lightly abraded with a thin wooden dowel to reduce the electrode-skin impedance to below 10 kΩ. The EEG data were recorded with a Neuroscan Synamps system (500 Hz sampling rate, 0.30–30 Hz bandpass filter). Participants were blindfolded and asked to keep their eyes closed during the various task conditions. A video monitoring system was used to ensure that participants remained still and that their hands were in resting position during EEG data collection.

### Auditory task

#### Study 1

A trial consisting of a sequence of three pseudo-randomly selected sounds from the same category (e.g., Hand sound—Hand sound—Hand sound) was presented at an inter-stimulus interval (ISI) of 1 s. No individual sound appeared twice within the same trial. Sixty trials (twelve from each sound category) were randomized and presented (inter-trial interval or ITI of 3 s) through circumaural headphones (Sony MDR XD 100) using Presentation software (NeuroBehavioral Systems). An additional five randomly interspersed sequences contained “oddball” events. These sequences contained a final sound that was out-of-category (e.g., Mouth sound—Mouth sound—Hand sound). Participants were instructed to click a computer mouse placed under their right hand when detecting such an oddball event. This task was necessary to ensure that participants were *actively* listening to all the stimuli. Control sounds were presented as triplets but were not used as oddball events. While analyzed for accuracy as a behavioral task, these oddball event trials were excluded from further analysis in order to avoid motor contamination of the EEG data. After the task, participants were given the opportunity to take a short break prior to the start of the motor task.

#### Study 2 modifications to auditory task

In the second study, the ISI was increased to five seconds and the ITI to seven seconds to increase the amount of time participants had to process the individual sound stimuli. The type of response to oddball events also differed, with covert responses in Study 2 to avoid the effects of motor actions. Like Study 1, sequences with “oddball” events were randomly interspersed in between the normal trials. However, instead of actively using a mouse to indicate the oddball events, participants were asked to covertly count the total number of oddballs presented throughout the auditory task. At the end of the block of trials, subjects were asked for the total number of oddball events counted. This task was necessary to ensure that participants were *actively* listening to all the stimuli. Control sounds were not used as oddball events. While analyzed for accuracy as a behavioral task, these oddball event trials were excluded from further analysis in order to avoid any type of motor contamination of the EEG data. After the task, participants were given the opportunity to take a short break prior to the start of the motor task.

### Motor task

In both Studies 1 and 2, participants were also asked to execute four actions associated with the sounds they heard. One was “zipping a zipper” (hand action) in which participants were provided a zippered jacket to put on prior to the start of the motor task. When cued, they were to move their hands from resting position on the armchair, zip the jacket up and down four times and return their hands to resting position. A second action, “ripping paper” (hand action) involved cueing the participant to move both hands from resting position on the armchair to the center where an experimenter would hand them a paper towel to rip. Participants would rip the paper towel three times, drop the scraps on the floor, and move their hands back to resting position. A third action, “sipping from a straw” (mouth action) involved cueing the participant by placing the straw to their lips and asking them to pretend drinking through the straw three times. The final action was “kissing” (mouth action) in which subjects were cued to make three kissing motions (i.e., purse and release their lips, thus making a smooching sound). During these actions, participants were blindfolded to prevent them from seeing their own actions, and circumaural headphones delivered white noise loud enough to prevent the participants from hearing the sound effects of their own actions. Experimenters used haptic cues [e.g., touching of hand] to prompt the participant to begin each action (the cues and actions were explained to the participant and practiced prior to EEG recording). Each of the four actions was performed twice, for a total of eight motor actions within a single block of trials. The order of the actions was determined through a random number generator, and was unique to each participant. In addition, the block of trials was repeated eight times for a total of 64 motor actions per subject. One experimenter delivered the haptic cues while a second confederate used a keyboard to send pulses via keyboard to the EEG computer demarcating the onset and offset of each action (see cues in Table 2).

**Table 2:** **Four Actions Performed During the Motor Task (MT)**.

**Motor action**	**Cue**	**Onset**	**Offset**
Zipping a zipper	Pull on jacket	Move from resting position	Return to resting position
Ripping paper	Touch hand	Move from resting position	Return to resting position
Sipping through a straw	Put straw to lips	Cue	Finish third “sip”
Kissing	Touch cheek	Cue	Finish third “kiss”

### Data analysis

Eye blinks and movement artifacts were digitally identified in the EOG recording and removed. Other types of EEG artifacts were also automatically and manually removed prior to analysis. Data were only analyzed if sufficiently “clean” EEG, with no movement or eye blink artifacts, were present. Between 10 and 30% of the data were removed for individual participants. For each cleaned segment the integrated power in the 8–13 Hz mu range was computed using a Fast Fourier Transform. Data were segmented into epochs of 2 s beginning at the start of the segment. Fast Fourier Transforms were performed on the epoched data (1024 points or 2046 ms). A cosine window was used to control for artifacts resulting from data splicing.

Suppression of the 8–13 Hz band in both the auditory and motor tasks was computed as the ratio of power in response to Action sounds, Non-Action sounds, and motor movements, relative to control sounds. A ratio was used to control for variability in absolute power as a result of individual differences in scalp thickness and electrode impedance, as opposed to absolute differences in electrical activity. Since ratio data are inherently non-normal as a result of lower bounding, a log transform was used for statistical analyses. A log ratio of less than zero indicates suppression whereas a value of zero indicates no suppression and values greater than zero indicate enhancement.

Accuracy on the Auditory Oddball Task was calculated as hits plus correct rejections divided by total number of blocks. For EEG suppression, an omnibus ANOVA was first run followed by distinct ANOVAs for midline (Fz, Cz, Pz), frontal (F3, F4, F7, F8), centro-parietal (C3, C4, P3, P4), temporal (T3, T4, T5, T6), and occipital (O1, O2) sites. Stimulus type (mouth sound, hand sound, motor mouth, motor hand, environmental sounds) and electrodes were used as within-subject factors with Greenhouse-Geisser corrections applied to the degrees of freedom and only the corrected probability values reported. Partial Eta scores (h^2^*_p_*) are also reported. Pairwise comparisons were conducted using Tukey's Honest Significant Difference (HSD), while a Bonferroni correction was applied to correct for multiple comparisons.

## Results

Trial lengths were longer in Study 2 (7 s ITI) than in Study 1 (3 s ITI) to allow for more processing of the stimulus, and the type of response to oddball trials differed, with overt responses used in Study 1 and covert responses in Study 2. Nonetheless, no statistical differences were found between the studies and therefore only the combined results are reported.

### Auditory identification task

During the Auditory Identification Task, participants were able to identify sounds with a high degree of accuracy within three times of listening to a sound. The specific results for Hand, Mouth, and Environment sounds were 95, 98, and 93%, respectively. During the oddball trials, participants were able to correctly detect the oddball event with 97% accuracy.

### EEG 8–13 Hz suppression

There was a main effect of stimulus type across all electrodes, *F*_(4, 220)_ = 63.2, *p* < 0.01, η^2^*_p_* = 0.535. Pairwise comparisons showed that all but audio hand vs. environmental sounds were highly significant (*p* < 0.01). There was also a main effect of electrodes, *F*_(16, 880)_ = 7.9, *p* < 0.01 and a stimulus type x electrode interaction, *F*_(64, 3520)_ = 5.99, *p* < 0.01.

**Figure 1:**
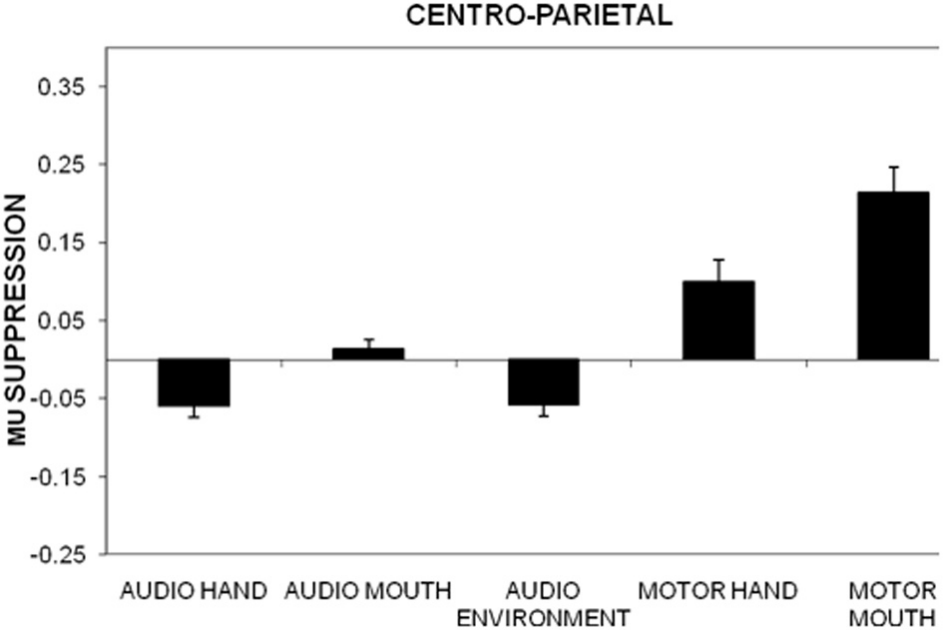
**Mu suppression over centro-parietal regions (C3, C4, P3, P4) in the action (hand, mouth) and non-action (environmental) auditory conditions as well as during the motor actions (hand, mouth)**. Mu suppression is determined as the log of the ratio between experimental condition and baseline condition. Error bars are standard error of the mean.

**Figure 2:**
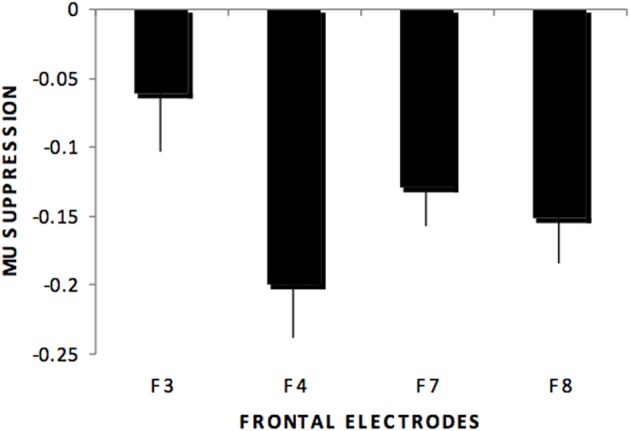
**Mu suppression across stimulus type at frontal regions (F3, F4, F7, F8) showing the stimulus type by electrode interaction in which greater mu suppression was recorded over right (F4) compared to left (F3) anterior frontal sites but not over more ventral anterior sites (F7, F8)**.

Individual ANOVAs for subset of the electrodes (frontal, centro-parietal, midline, temporal, occipital) showed similar results as the omnibus ANOVA, as shown in Figure 1 for centro-parietal sites. Likewise, frontal electrodes showed a main effect of stimulus type, *F*_(4, 220)_ = 48.8, *p* < 0.01, η^2^*_p_* = 0.470. Pairwise comparisons for this subset of electrodes indicated significant differences for all comparisons except the audio hand vs. environmental sounds. There was a significant stimulus type x electrodes interaction, *F*_(12, 660)_ = 3.29, *p* < 0.01, with greater suppression over right (F4) compared to left (F3) anterior frontal sites (see Figure 2). This difference occurred primarily for hand sounds.

**Figure 3:**
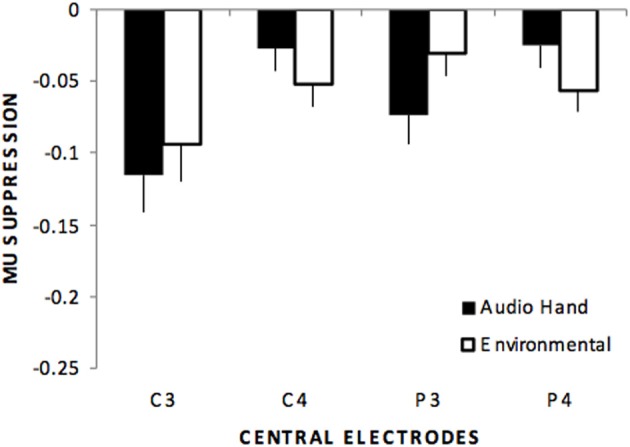
**Mu suppression over centro-parietal regions (C3, C4, P3, P4) showing that although no differences occurred between these sounds (audio hand vs. environmental), a stimulus type by electrode interaction showed that audio hand sounds exhibited greater suppression over left hemisphere sites (C3, P3) while environmental sounds exhibited greater suppression over right hemisphere sites (C4, P4)**.

Centro-parietal electrodes exhibited a main effect of stimulus type, *F*_(4, 220)_ = 31.6, *p* < 0.01, η^2^*_p_* = 0.365. Like the frontal electrodes, pairwise comparisons indicated significant differences for all comparisons except the audio hand vs. environmental sounds. There was also a main effect of electrodes, *F*_(3, 165)_ = 3.08, *p* < 0.05 with significantly more suppression over left (C3, P3) compared to right (C4, P4) hemisphere sites. A stimulus type x electrode interaction, *F*_(12, 660)_ = 3.09, *p* < 0.05 showed that in the audio hand and the environmental sounds conditions there was greater suppression compared to other conditions and more over left hemisphere for the audio hand condition and over the right hemisphere for environmental sounds (see Figure 3).

Midline electrodes showed a statistically significant main effect of stimulus type, *F*_(4, 220)_ = 55.0, *p* < 0.01, η^2^*_p_* = 0.500, with all pairwise comparisons showing significant differences. A main effect of electrodes, *F*_(2, 110)_ = 7.5, *p* < 0.01, showed greater suppression occurring at the central (Cz) compared to frontal (Fz) and parietal (Pz) sites. There was also an interaction between stimulus type and electrodes, *F*_(8, 440)_ = 9.5, *p* < 0.01 with audio hand showing the greatest suppression.

Temporal electrodes exhibited a statistically significant main effect of stimulus type, *F*_(4, 220)_ = 52.0, *p* < 0.01, electrodes, *F*_(3, 165)_ = 25.5, *p* < 0.01, and an interaction between stimulus type and electrodes, *F*_(12, 660)_ = 4.28, *p* < 0.01.

**Figure 4:**
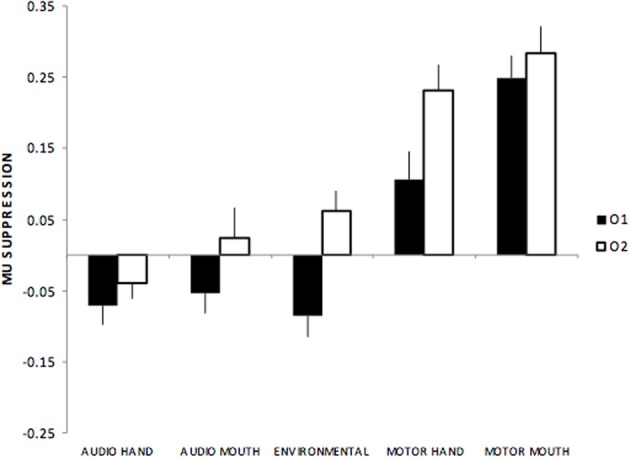
**Mu suppression over occipital regions (O1, O2) showing suppression over left (O1) while there was relative enhancement over right (O2) sites to environmental and motor actions**.

Occipital electrodes showed a main effect of stimulus type, *F*_(4, 220)_ = 42.1, *p* < 0.01, with all audio sounds (hand, mouth, environmental) showing suppression compared to more positive responses during motor actions. There was also a main effect of electrodes, *F*_(1, 55)_ = 15.2 *p* < 0.01, such that greater suppression was seen over left (O1) compared to the right (O2) hemisphere. Finally, there was an interaction between stimulus type x electrodes, *F*_(4, 220)_ = 4.2, *p* < 0.01 such that the greatest amplitude differences between left and right hemisphere sites occurred for environmental sounds (see Figure 4).

## Discussion

Results from this study show that EEG 8–13 Hz mu rhythms exhibit amplitude modulation not only during the performance of an action (synchronization) but also during hearing of action related sounds as well as non-action related sounds (desynchronization). Synchronization during action execution has been previously reported and while differences in the direction of modulation may reflect motor vs. mirroring processes, they may also involve increases in sensitivity to motivationally meaningful events (Pineda and Oberman, 2006). Differences in the spatial distribution of the mu suppression triggered by the action vs. the environmental sounds may reflect different neural sources, with action related sounds displaying a locus over left hemisphere in the anterior-posterior axis while non-action environmental sounds display primarily a right hemisphere locus.

A concurrent fMRI-EEG experiment (Arnstein et al., 2011) has shown that mu suppression during action observation and execution, as measured in the EEG over central electrodes (C3), directly correlated with BOLD increases in the dorsal premotor, and parietal cortex (posterior SI and adjacent posterior parietal lobe). Activity in the ventral premotor cortex was less correlated with mu suppression over C3. Because the exact same sound stimuli used here have been used previously in an experiment using fMRI (Gazzola et al., 2006), we can use the results of the fMRI-EEG study to link the results from the current EEG study and the past fMRI experiment. In particular, Gazzola et al. (2006) found that in the left hemisphere, the hand action sounds recruited the dorsal premotor and somatosensory cortex more than the environmental sounds [Figure S1 in Gazzola et al. (2006)], while the reverse was true for the right hemisphere. Here, we find the same lateralization pattern, with hand action sounds producing more mu suppression than environmental sounds over C3 (left) and the environmental sounds producing more mu suppression than the hand-action sounds over C4 (right). In addition, (Gazzola et al., 2006) found a somatotopic activation pattern, with hand action sounds and hand-action execution recruiting the dorsal premotor and mid-parietal cortex, while mouth action sounds and mouth action execution activated the ventral premotor cortex and the ventral-most parietal cortex. In the fMRI-EEG study, the dorsal but not the ventral premotor cortex, and the mid- rather than ventral parietal cortex reliably predicted mu suppression over C3. Accordingly, one would expect more mu suppression over C3 for the hand than mouth-actions. For the sounds, this was exactly what was found in the present experiment. For the action execution, mu suppression was not found for either of the action types, but the mu-power over C3 was lower for the hand than the mouth action execution. Accordingly, the two experiments using fMRI and EEG, respectively, find compatible results in terms of lateralization and somatotopical arrangement of the activation triggered using the same auditory stimuli.

These observations are also congruent with previous studies that have used different sounds but indicated that action sounds (those that are reproducible by the body) and non-action related sounds are processed by separate neural systems in the human brain (Pizzamiglio et al., 2005). More specifically Pizzamiglio et al. report that left posterior superior temporal and premotor areas appear to reflect action-related sounds, while bilateral areas in the temporal pole appear to respond to non-action related sounds. The present findings of a left hemisphere locus for action-related sounds underscore the fact that auditory aspects of mirroring, as reflected in the dynamics of the EEG mu rhythm, exhibits similar functional specialization inherent in processing auditory sounds with semantic meaning (Zahn et al., 2000). Furthermore, sounds associated with different effectors, e.g., hand compared to mouth action sounds, show distinct modulations of this rhythm. Hence, the data are consistent with the idea that biological sounds engage mirroring processes (both synchronization and desynchronization actions) in a manner similar to that which occurs during the observation and reproduction of motor actions (Pfurtscheller and Lopes da Silva, 1999; Neuper et al., 2006). The multi-sensory properties of the human MNS is thus assumed to help build a more accurate representation of sensorimotor activity from the visual, auditory, and other information embedded in the observation of other individuals. Whether these various aspects of the input are processed simultaneously or treated equally is left for future research.

Desynchronization or suppression of EEG rhythms has generally been interpreted as a correlate of an activated cortical area with increased excitability, while synchronization has been interpreted as a correlate of a deactivated cortical region (Pfurtscheller, 2001; Pineda, 2005). Mu rhythm oscillations in the present study were enhanced (meaning that the underlying neurons were less active and more synchronized) for mouth compared to hand stimuli, both while performing the movements in the Motor task and hearing the sounds in the Auditory task. In addition to fitting with the data of Gazzola et al. (Gazzola et al., 2006), using the same stimuli, the interpretation of these differences in mouth- and hand-related processing is also compatible with the findings of Pfurtscheller and Neuper (Pfurtscheller and Neuper, 1994) who reported that excitation of one sensorimotor area is typically accompanied by inhibition of a neighboring sensorimotor area as a result of lateral inhibitory connectivity. Consistent with this idea, mu rhythms recorded at central sites (C3, C4), which are located closer to the hand than mouth area in the motor strip, showed suppression to hand-related sounds and enhancement to mouth-related sounds. These center-surround effects, however, were asymmetrical since enhancements to mouth-related sounds were typically >20%, while suppressions to hand-related sounds were typically <10%. The basis for such an asymmetry is unclear although it appears consistent with greater suppression occurring to hand-related sounds at the C3 and C4 electrode sites. This difference cannot be attributed to task performance because no difference in counting of oddball control trials occurred for mouth- and hand-related sounds.

Participants understood and actively listened to the sounds as indicated by the results of the Auditory Identification Task and responses to the oddball events during the Auditory Task condition. The EEG findings are therefore not likely due to differences in perceptual or attentional factors but more consistent with the assumption that mirroring is reflected in the dynamics of the mu rhythm. That is, meaningful action sounds trigger greater mirroring as reflected in more mu suppression because they are embodied by the listener in order to understand them. This is supported by the different patterns of mu suppression to action (Mouth and Hand-based sounds) and non-action (Environmental) sounds. That is, greater suppression was recorded over the left hemisphere (especially over parietal areas) in response to action compared to non-action sounds but this is reversed over the right hemisphere. Thus, it is congruent with the idea that the mu rhythms (and the premotor to sensorimotor cortex connection) indexes auditory activity related to mirroring and that such a system exhibits lateralized processing as a function of the semantic associations with the sounds.

Studies 1 and 2 of the present work were designed to address the influence of motor preparation to report oddballs on the mu suppression results. We required participants in one study to make overt responses by clicking a mouse and in a separate study make covert responses by mentally counting the oddball trials. Both overt and covert responding requires motor planning. However, in one case it specifically involves an effector, such as the hand, while in the other it avoids such effector-based preparation. Since no significant differences occurred between the two studies, it suggests that EEG mu rhythms are either unaffected by the type of motor preparation or similar motor preparation occurs for both overt and covert responding.

When we observe another person moving, we only see the external consequences of their actions. To reproduce this action, we need instead to produce motor programs that produce a similar action. Clearly, the visual signals entering the eye during action observation are fundamentally different from the motor commands that need to be generated to perform a similar action. For one to map observed actions onto similar states in one self to understand or imitate the actions of others poses what has been called the correspondence problem in mirroring (Brass and Heyes, 2005). Specifically, how do observed movements actually map onto the observer's own motor system to enable everything from simple motor imitation to visceral discomfort upon seeing a queasy face? That is, how do we actually translate what we see into what we do (Brass and Heyes, 2005; Pineda, 2005)? In the visual domain, this correspondence problem is constrained by the fact that the observer can witness what body part the agent has used to perform the action. In the auditory domain, such information is lacking. When you hear the crunching of the soda can, it is impossible to know whether the left hand or the right was used. Perhaps it was the left or right foot used. Either is impossible to know with the given piece of sound information. Nevertheless, hand-action sounds and mouth-action sounds generated different patterns of mu suppression, which mirrors the relative amount of mu suppression during action execution, and previous fMRI studies have shown the existence of somatotopic brain activity (Gazzola et al., 2006) that allows classification as to which effector was used from sounds alone (Etzel et al., 2008). It has been proposed, that this somatotopy is the result of Hebbian learning: while we crush a coca-cola can with our right hand, we simultaneously perform the motor program, and hear (through what is called re-afference) the sound of this action. Through Hebbian learning, neurons in high-level auditory cortex that respond to the sound of this action then would enhance their synaptic connections with motor neurons in the parietal and premotor cortex that caused the action and with neurons in SI that sense the tactile consequences of performing such hand actions (Keysers and Perrett, 2004). Thereafter, listening to the sound would trigger, through these Hebbian associations, the motor programs corresponding to that action and the somatosensory representations of what such actions feel like. Because such motor programs and somatosensory fields are located more dorsal in the premotor, somatosensory and posterior parietal cortices than for mouth motor programs (Gazzola et al., 2006), the sound of such actions that we normally perform with our hands will trigger activity preferentially in these more dorsal regions in fMRI (Gazzola et al., 2006) and causing maximum mu suppression under C3 in the present study. While performing mouth actions like gurgling, we can hear and feel ourselves perform the action, and neurons in the high-level auditory cortex responding to the sound of actions we normally do with the mouth will wire together with the more ventrally located mouth motor and somatosensory representations in the premotor and parietal lobe. Accordingly, hearing such sounds will later trigger more ventral activity in the premotor and parietal lobe (Gazzola et al., 2006) and less mu suppression over C3. In support of the notion that brain activity in premotor and posterior parietal cortex is triggered by sounds as a result of Hebbian associations rather than inborn processes, Lahav et al. (Lahav et al., 2007) has shown that no premotor activity occurs to the sound of piano music in piano naïve listeners. However, a few hours of piano lessons, during which participants repeatedly experience the temporal contingencies between pressing piano keys and musical notes suffices to train new neural connections: after the training, piano music suddenly did trigger premotor activation in regions used to perform hand actions while listening to the learned piano melodies.

Environmental sounds such as the sound of a train passing are at first glance not considered body action-related, and it may seem odd that they should trigger any mu suppression at all. However, it is not unthinkable that they could be variably embodied, via Hebbian learning processes, in different individuals. Does John associate the sound of a train passing with the vibrations of his morning commute on the locomotive? Does Suzie associate the sound of a train passing with using her arm to move her electric train along the play tracks? The fact that such environmental sounds in the present study exhibit significant levels of mu suppression is consistent with this embodied argument for non-action stimuli. Nonetheless, much more research is needed to clarify these explanations.

### Conclusion

The findings of this study provide support for a mirroring system in the human brain that responds to specific sounds, as well as greater understanding of the distinctions and similarities between processing the auditory and visual aspects of mirroring. The patterns of mu suppression across cortical regions to different categories of sounds and to effector-specific sounds suggest differential engagement of this mirroring system in the human brain when processing different category of sounds and may offer a potential set of signals to explore for the development of a passive brain computer interface. Clearly, future studies are needed to specifically investigate that possibility, as well as the effects of auditory and motor tasks on special populations, such as autistic individuals, which in turn could provide insight on the degree of importance of the auditory MNS in social interactions and language.

### Conflict of interest statement

The authors declare that the research was conducted in the absence of any commercial or financial relationships that could be construed as a potential conflict of interest.
